# Auditory sensory range of male mosquitoes for the detection of female flight sound

**DOI:** 10.1098/rsif.2022.0285

**Published:** 2022-08-24

**Authors:** Toshiyuki Nakata, Patrício Simões, Simon M. Walker, Ian J. Russell, Richard J. Bomphrey

**Affiliations:** ^1^ Graduate School of Engineering, Chiba University, Chiba, Japan; ^2^ School of Life Sciences, University of Sussex, Brighton, UK; ^3^ Faculty of Biological Sciences, University of Leeds, Leeds, UK; ^4^ Pharmacy and Biomolecular Sciences, University of Brighton, Brighton, UK; ^5^ Structure and Motion Laboratory, Royal Veterinary College, Hatfield, UK

**Keywords:** mosquito, aeroacoustics, phonotaxis, flight, fluid dynamics, auditory physiology

## Abstract

Male mosquitoes detect and localize conspecific females by their flight-tones using the Johnston's organ (JO), which detects antennal deflections under the influence of local particle motion. Acoustic behaviours of mosquitoes and their JO physiology have been investigated extensively within the frequency domain, yet the auditory sensory range and the behaviour of males at the initiation of phonotactic flights are not well known. In this study, we predict a maximum spatial sensory envelope for flying *Culex quinquefasciatus* by integrating the physiological tuning response of the male JO with female aeroacoustic signatures derived from numerical simulations. Our sensory envelope predictions were tested with a behavioural assay of free-flying males responding to a female-like artificial pure tone. The minimum detectable particle velocity observed during flight tests was in good agreement with our theoretical prediction formed by the peak JO sensitivity measured in previous studies. The iso-surface describing the minimal detectable particle velocity represents the quantitative auditory sensory range of males and is directional with respect to the female body orientation. Our results illuminate the intricacy of the mating behaviour and point to the importance of observing the body orientation of flying mosquitoes to understand fully the sensory ecology of conspecific communication.

## 1. Introduction

Mosquitoes of medical importance—such as *Anopheles gambiae*, *Culex quinquefasciatus* and *Aedes aegypti*—use the sound generated from their wingbeats for intraspecific communication [1–3]. In these species, males initiate phonotactic flight when encountering the flight sound of females and modulate the wingbeat frequency in the vicinity of a sound source [4–6]. Female mosquitoes also modulate their wingbeat frequency during acoustic interactions [7–9]. These behaviours suggest that the ability of male mosquitoes to detect and localize a potential mate is mediated by the superposition of the flight-tone of both sexes. A deeper understanding of the acoustic behaviour of these mosquito species can enhance the efficacy of surveillance, provide new strategies for vector control and improve the quality of control programmes based on male mass release [2,10].

Acoustic communication requires at least one sound source, and complementary sensors. For mosquitoes, sound is generated by the flight apparatus [11]. Flapping wings generate periodic three-dimensional aerodynamic forces, and the fluctuations in the magnitude and direction of aerodynamic forces result in a dipole-like Gutin sound [12]. Among the possible range of sound-generating mechanisms, the tone produced by the flapping of mosquito wings is primarily produced by the mechanism associated with Gutin sound, just as a rotor blade produces a tone at the blade-passing frequency [11–13]. The wingbeat frequencies of male mosquitoes are higher than those of females; therefore, male flight-tones are higher in frequency than female flight-tones [4,9,14].

Mosquitoes detect the sound using the Johnston's organ (JO) that contains a radial arrangement of thousands of directionally sensitive sensory scolopidia in the antennal pedicel. The JO detects oscillations of the flagellum that are driven by particle motion in the air surrounding the antenna [15–17]. Thus, the JO is thought to be able to detect a signal regardless of its direction with respect to the flagellum. With the exception of [7,18], previous physiological studies showed the receptive bandwidth of the JO to be substantially lower than the wingbeat frequencies of the male or female mosquitoes but tuned to the frequency difference between the male and female flight-tones. Thus, the JO is tuned to respond to the difference tones—or distortion products—produced by the superposition of the fundamental frequencies of the flight-tones of a potential copulatory pair of mosquitoes [4,8,9,14,19,20]. Thus, males are not sensitive to a female's flight-tone unless he, too, is airborne and within earshot [4,8,9,14,19,20]. What that acoustic range may be is of crucial importance to the mating process but still the subject of debate.

While the behaviour of mosquitoes and the JO physiology has been investigated extensively within the frequency domain, the acoustic range of male mosquitoes for the detection of females is still not clear. Since particle velocities from dipole sound sources decrease steeply with the inverse cube of radial distance [15,21], it has been assumed that the hearing distance of the male against the female flight-tone is limited to close range, although male *Ae. aegypti* mosquitoes have been reported to respond to artificially loud sounds some metres away [22]. The pre-mating behaviour of mosquitoes has been investigated by measuring their kinematic or acoustic responses, but the auditory sensory range, as well as the behaviour of males at the initiation of phonotactic flights, are not well characterized because of the uncertainty in the soundscape generated by flying females.

In this study, we tested theoretical predictions of auditory range with a behavioural measurement of freely flying males. To begin, we quantified the three-dimensional distribution of particle velocity generated from female mosquitoes by aeroacoustic calculations based on aerodynamic simulations [23]. Next, we generated a hypothesis of the maximum distance at which a flying male should be able to perceive a flying female by combining our knowledge of the acoustic field of females with the sensitivity of the electrophysiological frequency response of the male JO measured in a previous study [4]. That is, we estimated the decline in particle velocity with distance from the emitter and determined an iso-surface corresponding to the maximum sensitivity of the JO. The estimated particle velocity iso-surface around a female mosquito represents the theoretical envelope within which males may be able to detect the female, and it is directional with respect to the female body orientation. Finally, we measured the three-dimensional trajectories of free-flying male mosquitoes responding to female-like pure artificial tone and identified a quantitative switch in their flight mode to a phonotactic trajectory. We presented the males with three levels of this simplified flight-tone and calculated the local particle velocity they would have received at the point in space at which they switched mode. In doing so, we quantified the sub-maximal hearing distances of male mosquitoes when they initiated phonotactic flight.

## 2. Material and methods

### 2.1. Mosquitoes

Colonies of *C. quinquefasciatus* Say (Muheza strain) were bred in controlled-environment chambers at 75% relative humidity, 26 ± 2°C and 12 : 12 light–dark cycles [4]. Larvae were reared on cat food pellets (Purina PetCare, Gatwick, UK) and adults were provided with 10% sugar solution *ad libitum*. Larval density was approximately 70 l^−1^ of water. Adult mosquitoes between 4 and 14 days post-emergence were tested during the first 3 h of the scotophase, when mating behaviour occurs under natural conditions. Adult *C. quinquefasciatus* were sourced for the kinematic measurements at the Royal Veterinary College, London. For the kinematic measurements, groups were maintained in microclimate chambers with controlled humidity (70–75%), temperature (26 ± 2°C) and 12 : 12 light–dark cycles. Males and females between 4 and 14 days post-emergence were tested.

### 2.2. Aeroacoustics model

#### 2.2.1. Kinematics and computational fluid dynamics analysis

The wing kinematics of *Culex* mosquitoes flying inside a transparent flight arena (330 mm × 330 mm × 230 mm) were measured using eight synchronized high-speed cameras (Photron SA3: 382 × 352 pixels, Photron Europe, Ltd) operating at 10 000 frames per second with an exposure time of 5 µs (electronic supplementary material, figure S1*a*,*b*). A group of four to eight individuals were released in the arena for each measurement and were kept in the arena for 1–6 h between 14.00 and 20.00 h until we filmed approximately 5 sequences or moved to the next group due to their low activity. The cameras were calibrated using custom-written, bundle adjustment code [24] for Matlab (Mathworks, Natick, MA, USA). We selected 15 male (12–62 wingbeats, 425 wingbeats in total) and 3 female sequences (15–42 wingbeats, 82 wingbeats in total) for kinematic analysis. Comparison of wing lengths during each sequence (using Tukey's honestly significant difference criterion) showed that male sequences comprise 12–15 individuals and the female sequences comprise 3 individuals. They were processed with a fully automated voxel-carving method (sometimes known as ‘convex hull reconstruction’) to reconstruct the coordinates of the wing outline [25]. Our kinematics dataset is composed of three angular positions about the wing hinge, and a fourth degree of freedom that describes twist along the wing's long axis. The base of the proboscis, the tip of the abdomen and the left and right wing roots were tracked manually and used to calculate the three-dimensional position and orientation of the mosquito body.

For our computational fluid dynamics (CFD) analyses, we used a dynamic flight simulator [26,27] that is based on the incompressible, unsteady, three-dimensional Navier–Stokes equations and can easily integrate the realistic morphology, kinematics and aerodynamics of insect flight. The simulator uses a multi-block, overset-grid method in which the computational domain is decomposed into a local grid, clustered near the wings and body, and a global Cartesian grid. We assumed symmetric motion of the left and right wings and applied a symmetric boundary condition at the sagittal plane of the body and background grid. More details about the kinematics and CFD analyses, including self-consistency tests, can be found in our earlier work [23]. The male CFD analyses have been published as part of that previous study, whereas the female kinematics and CFD are novel here. For both, the CFD output provides a time-resolved pressure distribution on the wing and body surfaces that can be used in an aeroacoustics simulator.

#### 2.2.2. Aeroacoustics

In order to simulate the distribution of sound pressure around the mosquitoes, we used an integral form of the Ffowcs Williams–Hawkings equation [28,29] based on Lighthill's acoustic analogy [30], which is a good approximation of the aeroacoustics for mosquitoes because of the small length scale of mosquitoes in comparison with the wavelength [31,32]. The volume inside a sphere with a radius of 150 times the mosquito wing length (female: 495.5 ± 12.9 mm, *n* = 3; male: 415 ± 12.9 mm, *n* = 15) was discretized into 41 × 41 × 41 nodes equally spaced along polar and azimuthal angles and clustered towards the centre of the sphere. The sound pressure at each node is calculated by numerically integrating the acoustic pressure from the CFD simulation. The surface pressure at each point was interpolated by an 11th order Fourier series to take into account acoustic delay (delay due to the distance between sound source and receiver). The sound pressure level (SPL) at the wingbeat frequency was calculated from the amplitude of the fitted sinusoidal wave at the wingbeat frequency. The size of the mosquitoes is roughly two orders of magnitude smaller than the typical distances between the females and males (order 10^0^ mm versus 10^2^ mm, respectively), and therefore the sound pressure *p* at each node can subsequently be converted into a particle velocity *v* emitted from a point source, as follows [33]:

2.1 $$v=\frac{\;p}{Z}\sqrt{1+{\left(\frac{c}{2\pi fr}\right)}^{2}},$$

where *Z* is the acoustic impedance of air, *f* is the wingbeat frequency, *c* is the speed of sound and *r* is the distance from the sound source.

The simulated particle velocity of females at a location 20 mm in front of their heads is 6.7 × 10^–5^ ± 1.2 × 10^−5^ m s^−1^ (*n* = 3). This value is comparable to the measured sound intensity of tethered female mosquitoes at 20 mm in front of their heads (5.7 × 10^−5^ ± 1.9 × 10^−6^ m s^−1^ [4]), which gives confidence in our aeroacoustic simulations.

The highest sensitivity of the JO can be found around the frequency of the male–female difference tone [4], so we can expect that the males in our behavioural experiments were able to respond to that frequency. The acoustic signal the male JO receives comprises the superposition sound from a nearby female (*A*_F_ and *ω −* *α* for signal amplitude and frequency, respectively) and the sound of his own flight apparatus (*A*_M_ and *ω* + *α*). Assuming that the tone from females has significantly lower amplitude than the male's own tone at the location of the male antennae ($A_{M}^{2}+A_{F}^{2}\approx A_{M}^{2}$ and $\left(1+{\left(A_{F}/A_{M})\right)}^{a}\approx 1+a(A_{F}/A_{M}\right)$), the sum of the signals can be written as follows:

2.2 $$\begin{array}{rl} A_{M}\sin(\omega +\alpha )t+A_{F}\sin(\omega -\alpha )t & =(A_{M}+A_{F})\cos\alpha t\sin\omega t+(A_{M}-A_{F})\sin\alpha t\cos\omega t\\ & ={\left(A_{M}^{2}+A_{F}^{2}+2A_{M}A_{F}\cos2\alpha t\right)}^{-1/2}\sin\left(\omega t+\mathrm{atan}\left(\frac{\left(A_{M}-A_{F}\right)}{\left(A_{M}+A_{F}\right)}\tan\alpha t\right)\right)\\ & \approx (A_{M}-A_{F}\cos(2\alpha t))\sin\left(\omega t+\mathrm{atan}\left(\frac{\left(A_{M}-A_{F}\right)}{\left(A_{M}+A_{F}\right)}\tan\alpha t\right)\right). \end{array}$$

Therefore, when the female sound is significantly quieter than the male sound—which will be the case because the female sound source is far further away from the male JO compared with the distance between the male's JO and his own flapping wings (*A*_M_ ≫ *A*_F_)—the amplitude of the difference tone (cos(2*αt*)) can be expressed by the amplitude of the female sound, *A*_F_. Based on this reasonable simplification, we estimate the maximum detectable range of the males using the simulated particle velocity at the wingbeat frequency of females at the location of the male antenna.

### 2.3. Behavioural experiment

In addition to the collection of free flight sequences for kinematic analysis, we also observed the flight paths of male mosquitoes when presented with a female-like flight pure tone in order to create a behavioural assay for their auditory sensory range.

#### 2.3.1. Behaviour arena

The behaviour of free-flying mosquitoes was recorded in a 30 cm cube arena (electronic supplementary material, figure S1*c*). The metal frame was covered with matt-black cotton fabric which is non-reflective to infrared (IR) light. The front side was covered by transparent acrylic to enable video recording of the arena interior. The top of the arena was covered with a removable dark plastic mesh to access to the interior and to allow the arena to be illuminated by two IR multi-LED floodlights positioned 1 m above the cage. The arena was placed on a vibration-damped table (Newport^®^, Irvine, CA, USA) inside a sound-attenuated booth (IAC^®^ Ltd).

#### 2.3.2. Acoustic stimulation

Artificially generated female-like tones were delivered into the arena from a sound source consisting of a 0.5 cm diameter plastic probe tip connected to an adapted Audio Technica® ATH A700AX speaker. The tip of the speaker was connected to the centre of one of the lateral cotton walls of the arena.

Sound from the speaker and flight-tones from the mosquitoes were monitored using a calibrated particle velocity microphone (Knowles^®^ NR-3158, Itasca, IL, USA) mounted approximately 40 mm above the sound source. Signals were pre-amplified 100-fold and digitized at 192 kHz using a Fireface^®^ UC sound card. Pure tone stimuli were generated using the sine wave function of Test Tone Generator 4.4 (EsserAudio^®^, 2011) and presented at three sound levels across trials.

#### 2.3.3. Video acquisition

For each trial, no more than two mosquitoes were placed inside the flight arena at the time of spontaneous circadian activity. Upon initiation of the flight of at least one mosquito, we started video recording and auditory stimulation at a given sound level. The experiment proceeded until we had phonotactic sequences for all the tested sound levels or the mosquitoes stopped flying. After this, the mosquitoes were replaced with new ones. Therefore, the trajectories at each sound level represent the results of different individuals from the same batch. With the exception of the IR illumination, all the lights inside the sound-attenuated booth were switched off. After 5–10 min of adaptation, the mosquitoes started to fly spontaneously. All behavioural experiments were conducted at approximately 30°C, which is within the range of temperatures of the natural habitat of the *C. quinquefasciatus* mosquitoes [34]. Video recordings at 25 fps were taken using two Smart IPC Hikivision^®^ cameras (1280 × 720 pixels) placed 1.5 m apart from the arena and aimed through its transparent face. Sequences were captured after the spontaneous initiation of flight and stored for subsequent analysis.

For each trial, a pure tone at 450 Hz was presented during 5 s to simulate the fundamental frequency of a female. The onset of the sound stimulus elicited phonotaxis and rapid frequency modulation (RFM) behaviour by the flying male [4]. At this sound frequency, male RFM response is extremely robust in the arena (electronic supplementary material, figure S1*c*) [4], so non-responses were rare and not considered. A small IR LED outside the arena was synchronized with the onset and offset of the sound as an indicator for the cameras. Three sound levels were tested, with particle velocities at the source of 1.8 × 10^−5^ m s^−1^, 1 × 10^−5^ m s^−1^ and 5.7 × 10^−6^ m s^−1^, which correspond to −10 dB, −15 dB and −20 dB relative to the reference particle velocity of the *C. quinquefasciatus* female flight [4]. Thus, the sound levels from the speaker are lower than those of female mosquitoes so, in this experiment, male mosquitoes would be predicted to initiate a phonotactic response nearer to the speaker than to female mosquitoes. Reducing or expanding the predicted response distance can be useful if working in a limited volume flight arena, or with cameras of limited resolution. Thirty phonotactic sequences were recorded, and three-dimensional flight trajectories were reconstructed from paired videos using the direct linear transformation method (DLTdv5 package [35]) for MATLAB (Mathworks, Natick, MA, USA). We calculated the SPL distribution within the flight arena by assuming the stimulus to be a point source of sound, a linear decrease with the distance [33], and no reflections from the cotton walls. The SPL distribution was converted into a particle velocity distribution using equation (2.1).

## 3. Results

### 3.1. Aerodynamics and aeroacoustics of female mosquitoes

**Figure 1.**
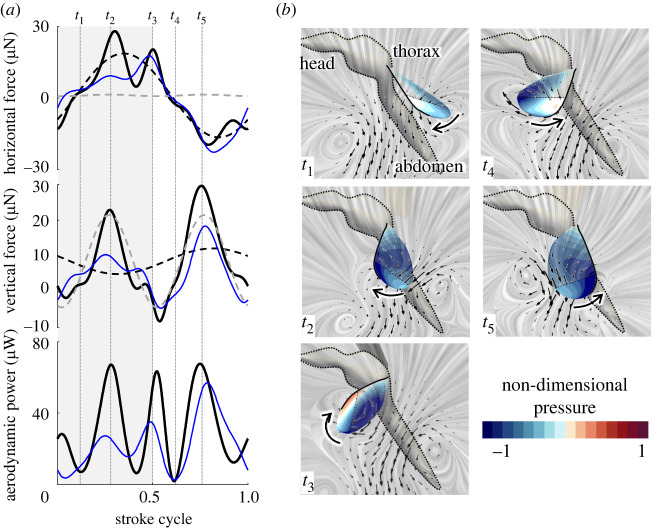
Aerodynamics of female mosquitoes from CFD analyses. (*a*) Time series of aerodynamic forces (top) and aerodynamic power (bottom). Black and grey broken lines represent the fitted sinusoidal curve at wingbeat frequency and second harmonic frequency. Blue lines represent the aerodynamic forces and power of a male [23]. (*b*) Surface pressure on the wing at *t*_1_–*t*_5_ in (*a*). Overlain are instantaneous streamlines visualized by the line integral convolution (grey) and flow vectors (black arrows) for vertical slices through the three-dimensional flow field at planes 0.6 or 0.75 wing length from wing base to show the flow field around the wing cross section (broken line).

We have previously published the kinematics and aerodynamics of male *Culex* mosquitoes [23] and can now compare between the sexes, using these results to calculate the acoustic signature of both. Females flap at a lower frequency than males (female: 475 ± 21 Hz, *n* = 3; male: 717 ± 59 Hz, *n* = 15). The wingbeat amplitude of females is greater than that of males (female: 50° ± 2°, *n* = 3; male: 39° ± 4°, *n* = 15), but is still markedly shallower than that of other insects [23]. The waveform patterns of flapping angles are similar to one another (electronic supplementary material, figure S2). Despite these variations in kinematics and the marked sexual dimorphism (wing length of female: 3.30 ± 0.09 mm, *n* = 3; male: 2.76 ± 0.09 mm, *n* = 15), our flow simulation results suggested that females exhibit aerodynamic mechanisms similar to those of male mosquitoes to fly [23]. To summarize, at the beginning of the downstroke, an attached trailing-edge vortex is generated with a strong suction region in the posterior portion of the wing as can be seen from the negative pressure (blue) in *t*_1_,*t*_4_ in figure 1*a*,*b* and a corresponding peak in aerodynamic force. So far, this mechanism remains unique to *Culex* mosquitoes and we can now report that it appears in the substantially larger and lower wingbeat frequency females as well as the males. High aerodynamic forces are achieved during each half-stroke by the leading-edge vortex mechanism seen not only in *Culex*, but also many other insects (*t*_2_,*t*_5_ in figure 1*a*,*b*) [36]. Rotational drag contributes to increased horizontal forces at the end of each half-stroke (*t*_3_ in figure 1*a*,*b*). As was seen previously in males, the highest force peak for both sexes is seen during the upstroke (*t*_5_ in figure 1*a*,*b*) because the leading-edge vortex and rotational drag effects are temporally blended (due to slight differences in up- and downstroke wing kinematics). There are four peaks in aerodynamic power: at the middle of each half-stroke and each stroke reversal. The aerodynamic power during downstroke is comparable with that during the upstroke for females, but it peaks during upstroke for males (figure 1*a*) [23]. The aerodynamic power at supination is also comparable to the peak during each half-stroke, which reflects the power required for rotational drag.

**Figure 2.**
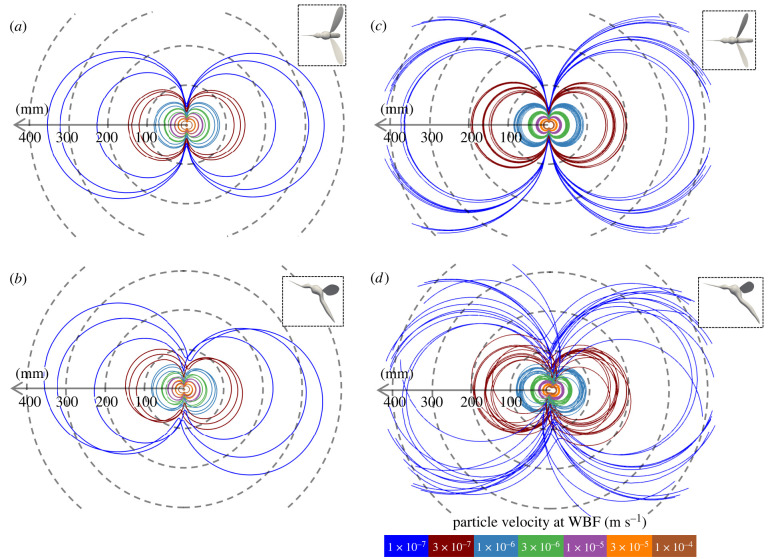
Aeroacoustics of mosquitoes. Top view (*a*,*c*) and side view (*b*,*d*) of the iso-lines of particle velocities emitted by females (*a*,*b*; *n* = 3) and males (*c*,*d*; *n* = 15) on the horizontal or vertical plane. The mosquito points to the left along the arrows. The insets show the top view and side view of mosquitoes for reference (not to scale).

Acoustic signatures during flight are also similar between males and females (figure 2) and strongly reminiscent of a dipole sound source [31]. The tone from both the male and female mosquitoes extends further along the anterior–posterior axis but less around the frontal plane (figure 2). Therefore, the mosquitoes are louder to the front or behind, while quieter above, below and to the side. The observed distribution is qualitatively consistent with measurements of tethered *Ae. aegypti* [37]. Because of the sagittal plane symmetry assumption of wing kinematics for our CFD simulations, the top view of the iso-surface is symmetric (figure 2*a*,*c*) but the side view is not. The particle velocity reaches furthest at an angle slightly tilted from the horizontal plane (figure 2*b*,*d*; female: 4.5° ± 4.5°, *n* = 3; male: 1.8° ± 11.4°, *n* = 15). Since the far-field sound is generated by the oscillation of surface pressure on the wings, the directionality in the aerodynamic force accounts for the three-dimensional profile of particle velocity. In other words, sideways forces are small and, consequently, so is the sound derived from the aerodynamic surfaces. As can be seen in figure 1*a*, the amplitude of the horizontal force at the wingbeat frequency is higher than those of the vertical force, which results in the higher horizontal oscillation of the sound pressure at the wingbeat frequency. In contrast, the second harmonics of the sound pressure should be stronger along the vertical axis because the second-order oscillations of vertical forces are larger than those of horizontal forces (figure 1*a*). The ratios of the vertical and horizontal forces are strongly affected by the stroke plane angle (female: 2.1° ± 5.9°, *n* = 3; male: 9.4° ± 8.9°, *n* = 15), which is close to horizontal in both females and males.

The three-dimensional distribution of particle velocities obtained from aeroacoustic simulations, combined with the sensitivity of the JO of male mosquitoes, allows us to predict the auditory sensory range of male mosquitoes. The maximum sensitivity of the JO of male *C. quinquefasciatus* is 10^−7^ m s^−1^ at the difference tone [4]. Therefore, the iso-surface of the particle velocity at 10^−7^ m s^−1^ (blue lines in figure 2*a*,*b*) represents the theoretical prediction of the three-dimensional auditory sensory range of the male. The iso-surface extends in the anterior–posterior direction and is waisted close to the mosquito. The maximum distance from the female that her tone could be heard is 312.3 ± 75.3 mm (*n* = 3) (figure 2*a*,*b*) in the anterior–posterior direction. Counterintuitively, considering their smaller body size, the particle velocity iso-surface for males reaches further than that of females (425.2 ± 77.9 mm, *n* = 15, *p* = 0.035; figure 2). Since the intensity of the Gutin sound is proportional to the body mass and wingbeat frequency in the far field [11], the higher frequency of males outweighs the effect of their smaller size and, therefore, the male sound reaches slightly further. However, because the male JO is sensitive to the male–female difference tone [4], this male sound would not be detected by other males at this distance.

### 3.2. Male behaviour in response to a simplified female flight-tone

**Figure 3.**
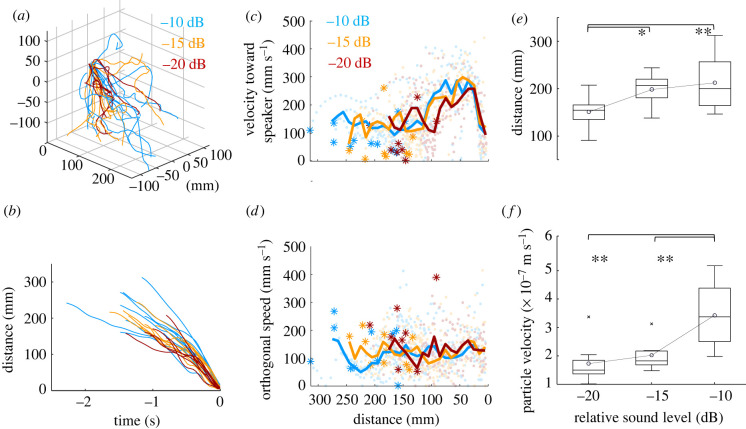
Phonotactic flight of males. (*a*) Flight trajectories of male mosquitoes responding to the tone (450 Hz) of −10 (cyan, *n* = 12), −15 (orange, *n* = 10) and −20 dB (red, *n* = 8). The sound level is relative to the reference particle velocity (average particle velocity of female flight: 5.7 × 10^−5^ m s^−1^) at 20 mm from the speaker. Speaker is located at the origin (black dot). (*b*) Time series of the distance towards the speaker where arrival at speaker is time zero. (*c*,*d*) The velocity towards the speaker (*c*) and the speed orthogonal to the direction from a male to the speaker (*d*) against the distance towards the speaker. The solid lines represent the velocity averaged over the individuals. Light-coloured dots represent the velocity, speed against the distance towards the speaker at every frame and individual. The asterisks represent the distance where phonotaxis was initiated. (*e*,*f*) Distance towards the speaker and the particle velocity at the onset of phonotactic flight. *Post hoc* pairwise ANOVA shows the degree of significance for groups found to be different under the Tukey criterion (*p < 0.05; **p < 0.01).

Free-flying male mosquitoes approached the sound source on presentation of the female-like pure tone, reducing the distance monotonically (figure 3*a*,*b*). During phonotactic flight, males flew with a relatively constant-oriented velocity of 100–150 mm s^−1^ towards the speaker while maintaining a similar speed in directions orthogonal to the speaker bearing (figure 3*c*,*d*) at 90.6–271.2 mm (−10 dB), 132.4–244.3 mm (−15 dB) and 73.6–207.8 mm (−20 dB). At a later stage of phonotaxis, at approximately 100–120 mm from the speaker, they accelerate further and the mean speed towards the speaker reaches 250–350 mm s^−1^, while the orthogonal speed still remains constant. This late acceleration towards the speaker was observed for all tested sound levels.

When the sound level at the speaker is increased from −20 to −15 dB, the distance at which the mosquitoes initiate phonotaxis also increases (figure 3*e*, *p* = 0.047). However, initiation of phonotaxis is invariant with respect to the calculated particle velocity (figure 3*f*, *p* = 0.243). The minimum particle velocity at which phonotaxis is initiated is consistently around 10^−7^ m s^−1^, with the absolute distance varying depending on the sound level setting of the speaker. At the highest stimulus sound level of −10 dB, the particle velocity exceeds the lower threshold of JO sensitivity at all points within the flight chamber (2 × 10^−7^ m s^−1^ at 310 mm from the speaker, based on equation (2.1)) and can be thought of as a control measurement, where phonotactic response would always be expected. This limitation of the chamber size explains why there was no significant difference in the distance of the initiation of phonotaxis at −10 dB compared to −15 dB (which already covers most of the chamber; figure 3*e*, *p* = 0.174), and the particle velocity experienced at the instant of phonotactic response was significantly greater at the −10 dB level than the −20 or −15 dB levels (figure 3*f*, *p* = 1.2 × 10^−3^, 6.4 × 10^−3^). Even so, the similar ranges of particle velocities at −20 and −15 dB sound levels suggests that the male's JO minimum threshold at female wingbeat frequency, or the difference tone (see equation (2.2)), to initiate the phonotaxis can be estimated as 10^−7^ m s^−1^.

### 3.3. Auditory sensory range

**Figure 4.**
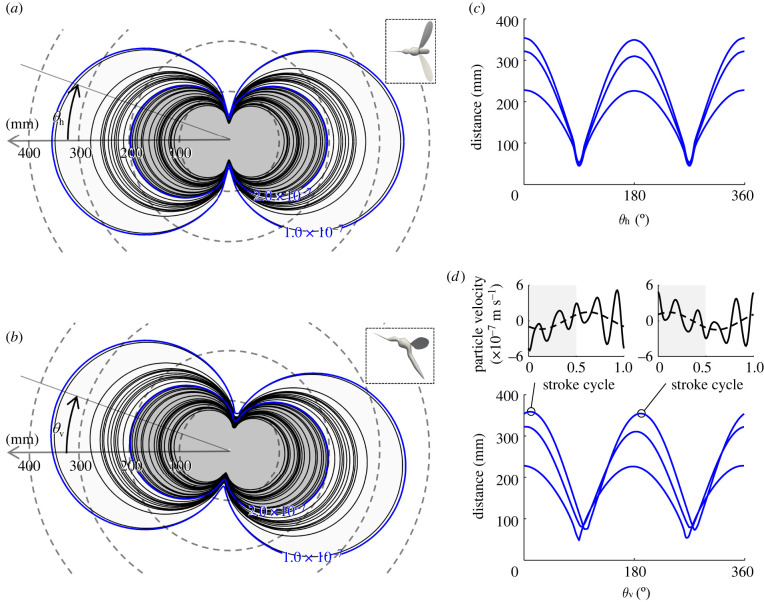
Auditory sensory range of males with respect to a female pointing left at the centre (see insets). Top view (*a*) and side view (*b*) of the iso-lines of estimated particle velocity on the horizontal or vertical plane (black). The iso-lines of 1.0 and 2.0 × 10^−7^ m s^−1^ are shown for reference (blue). The minimum threshold from the behavioural experiment was 1.0 × 10^−7^ m s^−1^ (figure 3*f*). Insets show the top view and side view of mosquitoes for reference (not to scale). (*c*,*d*) Effects of horizontal (*c*) and vertical (*d*) angles, *θ*_h_ and *θ*_v_ in (*a*,*b*), on the distance between the iso-surface of particle velocity (10^−7^ m s^−1^) and a female mosquito. Time series of the particle velocity at the direction with maximum distance in front (left) and at the back (right) of the female are shown in the top row in (*d*).

We used the acoustic signature of the females to identify iso-surfaces of the particle velocities estimated from the behavioural experiments. These are visualized around the female model in figure 4*a*,*b*, which represents our estimate of the auditory sensory range of males as predicted from our behavioural assay. As described above, the iso-surface of the particle velocity is directional with respect to the female orientation and so, then, is the sensory range. The distance at which male mosquitoes initiate phonotaxis depends on the intensity of the tone produced by female mosquitoes. Responding to the tone from the loudest female individual, males can initiate phonotactic flight at a maximum of 199.0 ± 59.9 mm (*n* = 30) from a female, and the individual with the largest sensory range observed in this study responded at 352 mm (figure 4*b*). If we travel along the iso-surface (which resembles a dumbbell, or two touching spheres) in different directions, the maximal detection distance reduces sinusoidally towards the frontal plane in both horizontal and vertical planes (figure 4*c*,*d*).

We can look closer at the waveform given by the particle velocity time history at any given location. If we observe the position where the particle velocity of 10^−7^ m s^−1^ reaches the farthest distance from the female to the front and rear, we see that they are out of phase relative to one another (figure 4*d*). Using synchronous recordings from several microphones, it was confirmed that the waveforms of sound pressure were also reversed in tethered *Ae. aegypti* [37]. This phenomenon occurs because the direction of the sound pressure generated by the aerodynamic pressure on the wing surface is reversed. In other words, an observer behind the animal will see the lower pressure of the upper surface of the wing during the downstroke, while an observer ahead of the animal will see the higher pressure on the lower surface; these surfaces are reversed during the upstroke when the wing has flipped over and reversed direction.

## 4. Discussion

The behaviour and physiology of mosquitoes have been of great interest because of their importance in global human and animal health. The frequency response of the JO auditory physiology had previously been reported, including in the context of how the male JO operates in the presence of both male and female flight-tones [4,8,9,14,19,20]. However, details of how neural encoding of the difference tone manifests in the male's behavioural response in terms of modified flight trajectories and its maximum range have hitherto remained unknown, or at least unconnected. Here, by presenting flying males with simplified female flight-tones at different sound levels, we could measure the stereotypical response of mosquitoes to the female tone in a repeatable manner. Moreover, we could identify the sensory range measured as the particle velocity limit within which the male JO must be able to detect females from the behavioural measurement. We shed light on the black box of this behavioural assay by using high fidelity fluid dynamics and aeroacoustic simulations to calculate the particle velocity distributions around females, which would have been difficult if solely relying on behavioural measurements of flying mosquitoes. The aerodynamic and aeroacoustic models of females in this study link the underpinning physics of flight to the auditory neuroscience of mosquito antennae. We have, therefore, enabled a quantitative estimate of the envelope of the male's auditory sensory range for male–female interaction, bridging the gap between trajectory-based behaviours and JO thresholds measured by electrophysiology. This approach appeared to be justified because predictions closely matched behavioural observations.

### 4.1. Sensory threshold and phonotactic flights of males

Our behavioural sensory range measurements suggest, as expected, that males initiate a phonotactic response at greater distances from the sound source when the presented tone is louder. When the distances are converted to the local particle velocity, the males respond at similar levels of the flight-tone, except for the presented tones of −10 dB which exceeded the dimensions of the whole chamber, i.e. everywhere within the chamber had particle velocities in excess of threshold, and, thus, our behavioural assay was incapable of presenting the extreme distances at which they could potentially respond. The lowest particle velocity for the initial phonotactic response is at around 10^−7^ m s^−1^, which is a good match with the most sensitive recordings of neural responses from the JO (1–2 × 10^−7^ m s^−1^; 5 dB above the noise floor) of male *C. quinquefasciatus* at the difference tone [4] and accords with the JO sensitivity of 1.5 × 10^−7^ m s^−1^ [9] and behavioural sensitivity to particle velocity of less than 5 × 10^−7^ m s^−1^ [38] of *Anopheles* mosquitoes.

Once males perceive the flight-tone of a female, they fly towards the sound source, reducing the distance to the sound source monotonically. Males can localize a female by the radially distributed sensory units of the JO [17]; each unit responds selectively to a sound coming from a restricted angular range and has a corresponding antiphase unit that responds over a similar dynamic range, but in the opposite direction. They start to accelerate at around 100–120 mm from the sound source in spite of the difference in the sound level. When landing on a wall, male mosquitoes have been reported not to prepare for landing but to absorb the impact with their proboscis and front legs [39], yet in the phonotactic flight observed in this study, the mosquitoes decelerated as they approached the sound source. Similar behaviours have been observed in parasitic flies that perform phonotactic flights in response to the pressure component of acoustic cues in search of hosts. *Ormia ochracea* [40], for example, can accurately locate a host based on sound alone. Their phonotactic trajectories are always curvilinear and horizontal during the cruising phase. At the last approximation, one or more velocity peaks occur, which coincide with trajectory positions at which the fly passes over the sound source and swiftly turns back to enter their diving spiral. Male mosquitoes also depend on sound alone to perform the phonotactic flight. In the swarm, male mosquitoes are in competition to catch female mosquitoes before other males. It is possible that the observed acceleration in their final phonotactic approximation may be necessary as the position of the source becomes more certain as he approaches ever closer. The sensorimotor mechanism behind this two-phase stereotypical phonotactic flight is unknown, but it may be elicited by the relative increase of the sound level with the approach towards the sound source.

### 4.2. Directional auditory sensory range of male

Our aeroacoustic simulation demonstrated that female tones are louder to the front and rear, which is a similar pattern to the male [31]. This observation suggests that a male could perceive a female from further away (up to approximately 350 mm) if the female is oriented pointing towards or away from him. However, this distance is greatly reduced if the male and female are oriented parallel, such as might be the case when flying in a lateral position with respect to the female, and in a similar direction. Behavioural observation of the mating between free-flying male and tethered female *Ae. aegypti* demonstrated that the male tends to approach the female from the front [6]. This behaviour could well be an effect of clear directionality in the acoustic profile. When females fly freely, this phenomenon provides an incentive for males to patrol or sweep the airspace to increase the likelihood of flying fore or aft of a female. At the same time, the female becomes more detectable if she changes body direction frequently, because it is unlikely that the male stays exactly in front of or behind her when both of them are flying freely. Under natural conditions, male *Culex* mosquitoes swarm at a location, and females are attracted to the swarm [41–45]. As the female enters the swarm, her heading presents the anterior view of her acoustic signature, aiding her potential mates to hear her approach from maximal range (figure 4), beginning with those on the near side of the swarm. Moreover, the general hum of the male swarm will not mask her approach from the male JOs, since they are more sensitive to the male–female difference tone [4,5,8,9,14,19,20].

There is a discrepancy in the literature about the range at which the male mosquitoes can detect females. It has been recently reported that the male *Ae. aegypti* can hear at metres away from the sound source [22]. Functionally, it is possible that sound pressures could vibrate in the wings (or nearby substrates) of the mosquito, which could be detected by the JO, but these hypotheses are untested. Additionally, flight initiation was used as a behavioural measure of sound detection, regardless of the type of acoustic stimulus delivered; however, it is unknown if this response—like an increase in flight speed [46]—is attractive or repulsive. In *C. quinquefasciatus*, the presentation of female-like tones did not elicit RFM behaviour (and indeed flight initiation) in resting mosquitoes [4]. Although Menda *et al.* [22] proposed that male mosquitoes can hear as low as 31 dB (a whisper) at 3 m away from the sound source, they may only respond behaviourally to nearby sounds.

The results in this study offer insight into the mating behaviour of mosquitoes and suggest guidelines for the design of future experiments. Mosquito trajectories can be reconstructed in a laboratory or the field by assuming that individuals are single points in space [47–50]. However, the directionality of the male's sensory range with respect to the body orientation of a female (figure 4) means that the orientation of males with respect to female orientation is also an important factor when considering acoustic signals that males may be receiving. Due to the limited resolution of cameras when observing a wide field of view, it is often difficult to measure body orientation at the same time as long flight trajectories. However, the measurement of female body orientation in addition to male flight trajectories prior to copulation would deepen our understanding of mosquito mating behaviour.

For surveillance, vector control and male mass release programmes, it is essential to know at what distances mosquito auditory detection and interaction take place. Outcomes from the current study reveal that, for male *C. quinquefasciatus* mosquitoes, this is less than 0.5 m from natural acoustic attractants (and is limited by the response properties of the JO and depends on the orientation of the male mosquito to the female sound). We can speculate that there should be a period between detection of sound and phonotactic initiation in which the males would orient and position optimally in relation to the sound source. This stage of initial approximation and orientation of the male is not yet described nor studied, and it was only very vaguely defined as the latency stage of the RFM behaviour [4].

Additionally, acoustic lures and traps should be designed to attract mosquitoes from a short range and genetically manipulated mosquitoes should be able to respond phonotactically to females at distances of up to 0.5 m away to be competitive with naturally occurring populations. This research area has currently been mainly focused on capturing male mosquitoes to monitor sterile male release programmes by broadcasting the fundamental wingbeat frequency of conspecific females using modified traps [3]. Understanding mosquito behaviour and response to acoustic cues can lead to better surveillance strategies and implementation of sound-based traps in the field [3].

Our results could, for instance, inform the design of better active mosquito traps which require powered suction fans. Much, if not all [3], considerations of sound level on the design of the acoustic traps are defined in SPL. Although sensible from a practical point of view (SPL is much easier to measure than particle velocity), this approach is not an accurate representation of the way mosquitoes detect sound. By defining a sensory volume for phonotactic activation in particle velocity, we hope this will lead to trap designs with increased acoustic attractiveness for male mosquitoes. An optimal sound level is crucial because, for some mosquito species, acoustic trap attractivity decreases at extreme (low and high) SPL levels [3].

Given the importance summarized above, the evaluation of auditory sensory range of other vectors, such as *Anopheles* and *Aedes* mosquitoes, is also useful, and there is no fundamental impediment to reproduce this study in those species. As in *Culex*, males of these genera have been shown to display a phonotactic response to female-like frequency tone [5,6,51].

## Data Availability

Datasets underpinning the current study are available from the Dryad Digital Repository: http://doi.org/10.5061/dryad.t76hdr83r [52]. Electronic supplementary material is available online at [53].
